# Pan-immune-inflammation value as an independent prognostic marker in patients with brain metastases

**DOI:** 10.3389/fonc.2025.1718288

**Published:** 2026-01-06

**Authors:** Jiacheng Li, Menghan Liu, Yingtong Liu, Zheran Liu, Jiazhen Liu, Yuping Xie

**Affiliations:** 1 College of Medical and Life Sciences, Chengdu University of Traditional Chinese Medicine, Chengdu, Sichuan, China; 2 Department of Biotherapy, Cancer Center, West China Hospital, Sichuan University, Chengdu, China; 3 Sichuan Provincial Key Laboratory of Nuclear Physics and Medical Research, Sichuan University, Chengdu, China; 4 Department of Nephrology and Nephrology Institute, Sichuan Provincial People’s Hospital, School of Medicine, University of Electronic Science and Technology of, China, Chengdu, China; 5 Department of Oncology, West China Fourth Hospital, Sichuan University, Chengdu, China

**Keywords:** brain metastases, nomogram, pan-immune-inflammation value, prognosis, retrospective analysis

## Abstract

**Background:**

Systemic inflammation and immune dysregulation are recognized as key determinants of cancer progression and survival. The pan-immune-inflammation value (PIV), a hematologic-based composite biomarker, may reflect the host’s immune-inflammatory status. Its prognostic significance in brain metastases (BM), however, remains undefined.

**Methods:**

In a single-center retrospective cohort, 3,856 consecutive patients with radiologically confirmed BM diagnosed between 2013 and 2021 were included. PIV was calculated as neutrophil count × platelet count × monocyte count, divided by lymphocyte count. All blood cell counts were recorded in units of 10^9 cells per liter. Complete blood counts were taken within 7 days before the start of treatment. The optimal PIV cut-off, derived using maximally selected log-rank statistics, defined low and high PIV groups. OS was analyzed using multivariable Cox models adjusted for age, performance status, number of BM and extracranial metastases. A PIV-augmented GPA nomogram was developed and internally validated with bootstrap resampling. Time-dependent concordance indices, calibration and integrated discrimination improvement (IDI) were used to assess model performance. Subgroup and sensitivity analyses examined robustness across systemic and local treatment modalities, primary tumor types, sex and alternative PIV parameterizations.

**Results:**

The PIV cut-off separated 1,570 patients with low PIV and 2,286 with high PIV. High PIV was associated with worse OS and remained independently prognostic (hazard ratio 1.40; 95% confidence interval 1.29–1.52; p < 0.001), with consistent effects across treatment modalities, primary tumor types and sex. Alternative cut-offs and modeling PIV as a continuous variable (per 1-standard-deviation increase) produced effect estimates similar to the primary analysis. Adding PIV to the GPA modestly improved discrimination and increased IDI by 0.010 (95% confidence interval 0.006–0.015; p < 0.001); the PIV+GPA nomogram showed good 1-year calibration.

**Conclusions:**

PIV is an independent prognostic factor for OS in BM patients. Incorporating this marker into the Graded Prognostic Assessment modestly improves risk stratification and supports an accessible nomogram for individualized survival prediction. External prospective validation, including longitudinal assessment of the pan-immune-inflammation value and integration with molecular and imaging markers, is needed before routine clinical implementation.

## 1 Introduction

Cancer remains a leading cause of mortality worldwide (1, 2). Brain metastases (BM), which occur in 10-40% of patients with advanced malignancies, represent one of the most devastating complications of systemic cancer progression (3, 4). The development of BM reflects a selective and biologically regulated metastatic process in which circulating tumor cells must survive hematogenous dissemination, adhere to cerebral endothelium, traverse the blood-brain barrier (BBB) (5), and interact with astrocytes (6), microglia, and infiltrating myeloid cells that collectively establish an immunosuppressive and tumor-permissive niche (7). These mechanisms underscore that BM is not merely a late manifestation of systemic disease but a biologically distinct metastatic entity (8).

Prognosis in BM is highly variable, influenced by primary tumor histology (9, 10), intracranial and extracranial disease burden, prior treatments, and heterogeneous responses to contemporary systemic therapies (11, 12). The rapid evolution of targeted agents and immunotherapies has further increased prognostic variability, making survival prediction increasingly challenging in routine practice. To provide standardized prognostic assessment, the Graded Prognostic Assessment (GPA) was developed and incorporates age, performance status, and extracranial disease burden (13). Although widely adopted, the GPA is derived exclusively from traditional clinical variables and therefore lacks biological indicators that reflect systemic inflammation, immune competence, or tumor-host interaction (14). As a result, its discriminative performance remains moderate (15), particularly in the modern treatment era, and it does not fully capture the biological processes that shape outcomes in BM (16).

Systemic immune and inflammatory activation has emerged as a key determinant of metastatic progression, treatment responsiveness, and survival across cancer types (17). Neutrophils, monocytes, platelets, and lymphocytes participate in tumor immune evasion, metastatic niche formation, and regulation of antitumor immunity (18–22). Biomarkers reflecting these compartments, such as the neutrophil-to-lymphocyte and platelet-to-lymphocyte ratios, have demonstrated prognostic value in multiple malignancies (23, 24), highlighting the potential of immune-inflammation indices to complement clinical prognostic models.

The pan-immune-inflammation value (PIV) is a composite biomarker that integrates neutrophils, monocytes, platelets, and lymphocytes, thereby providing a multidimensional representation of systemic inflammatory and immune status (25). Elevated PIV has been consistently associated with poor prognosis in cancers including lung cancer, melanoma, and breast cancer (26–28). However, despite its biological relevance, the prognostic utility of PIV has not been established in patients with BM, a population in whom systemic inflammation may be particularly reflective of tumor-host interactions due to the immunologically unique intracranial microenvironment.

Given the limitations of existing prognostic tools and the central role of inflammation in metastatic biology, we hypothesized that PIV could improve survival prediction in BM. We therefore conducted a large retrospective study of 3,856 patients to evaluate the prognostic value of PIV and to develop an integrated nomogram combining PIV with established clinical variables for individualized risk stratification.

## 2 Materials and methods

### 2.1 Study design and ethics

We conducted a retrospective cohort study of patients with brain metastases treated at West China Hospital, Sichuan University, from December 2013 to August 2021. Eligible cases were identified using ICD-10 code C79.3. Demographic, clinical, imaging, and laboratory data were extracted from electronic medical records, and vital status was ascertained through the Household Registration Administration System. The study was approved by the Institutional Review Board of West China Hospital (No. 2022127); the requirement for informed consent was waived owing to the retrospective design. The study adhered to the principles of the Declaration of Helsinki.

### 2.2 Patients

Eligible patients had a confirmed primary solid tumor and radiologically verified BM at initial presentation or relapse, assessed by CT, contrast-enhanced CT, MRI, or PET-CT, with or without histopathologic confirmation. Patients with erroneous registry identifiers, missing survival outcomes, or incomplete baseline PIV-defining hematologic data (neutrophil, platelet, monocyte, or lymphocyte counts) were excluded. Among 4,166 screened patients, 3,856 with complete PIV data were included in the final analysis. Follow-up was measured from the date of BM diagnosis until death or last contact, and survivors were censored at the last follow-up.

### 2.3 Variables and outcomes

The primary exposure was the PIV, calculated as (neutrophils × platelets × monocytes)/lymphocytes (all in ×10^9^/L) (13). Baseline hematologic counts were obtained within 7 days of BM diagnosis and prior to the initiation of any treatment for brain metastasis, including systemic chemotherapy, targeted therapy, radiotherapy, or other anti-tumor interventions. PIV was analyzed as a continuous variable and dichotomized using maximally selected log-rank statistics. Prespecified covariates included age (<50, 50-60, >60 years), Karnofsky Performance Status (KPS; <70, 70-80, 90-100), extracranial metastases (present/absent), and number of BM (single, 2-3, >3). Treatment modality (chemotherapy, targeted therapy, radiotherapy) and sex were recorded for subgroup analyses. The primary endpoint was overall survival (OS), defined as time from BM diagnosis to death from any cause; patients alive at last contact were censored.

### 2.4 Subgroup analysis

To examine the consistency of PIV’s prognostic value across clinical contexts, patients were stratified by treatment modality (chemotherapy, targeted therapy, radiotherapy), primary tumor type, and sex. Within each subgroup, univariate and multivariable Cox models using the same covariate set as the main analysis were fitted. Interaction terms between PIV and each subgrouping variable were tested to evaluate whether the prognostic effect differed across strata. Hazard ratios (HRs) with 95% confidence intervals (CIs) were reported. Results for the sex-based and primary-cancer-based analysis are presented in the **Supplementary Materials**.

### 2.5 Model comparison

Model performance was compared across the GPA model, PIV alone, and the combined GPA+PIV model. Discrimination was evaluated using time-dependent concordance index (C-index) curves over the follow-up period. Incremental predictive value was quantified with the Integrated Discrimination Improvement (IDI), with statistical inference based on perturbation resampling (1,000 iterations). Calibration was assessed by plotting predicted versus observed 1-year survival using bootstrap resampling with 500 iterations.

### 2.6 Sensitivity analyses

To assess the robustness of the primary findings, two sets of sensitivity analyses were performed. First, Cox proportional hazards models were repeated using the median PIV value as an alternative dichotomization threshold to evaluate whether the observed associations were dependent on the choice of cutoff. Second, PIV was modeled as a continuous variable in multivariable Cox regression to determine whether the prognostic effect persisted without categorization. Concordance of effect estimates across these analyses was interpreted as evidence of stability.

### 2.7 Statistical analysis

Continuous variables were summarized as mean ± SD or median (IQR) and compared using the t test or Mann-Whitney U test, as appropriate; categorical variables were compared using χ² test. OS was estimated with Kaplan-Meier methods and compared using log-rank tests. Cox proportional hazards models were applied for univariate and multivariable analyses. Age, KPS, number of brain metastases, and extracranial metastases (ECM) were adjusted in multivariate cox analysis A prognostic nomogram was developed from independent predictors (including PIV). and internally validated with 200 bootstrap resamples. Model performance was evaluated using the optimism-corrected C-index (optimism-corrected) and 1-year calibration. Two-sided p < 0.05 was considered statistically significant. All analyses were performed in R version 4.4.0 (R Foundation for Statistical Computing) with standard packages (e.g., survival, rms, survminer, survIDINRI).

## 3 Result

### 3.1 Patient characteristics and PIV cutoff determination

A total of 3,856 patients with BM were included in this retrospective analysis (**Table 1**). The mean age was 56.8 years, and 43.7% were male. Lung cancer was the predominant primary tumor, followed by breast cancer and nasopharyngeal carcinoma. Most patients presented with multiple brain lesions, and 69.1% had extracranial metastases. Using maximally selected log-rank statistics, the optimal PIV cutoff was determined as 225.589 (**Supplementary Figure S1**), stratifying patients into low (n=1,570) and high (n=2,286) PIV groups. Compared with the low PIV group, patients with high PIV were older, less frequently male, exhibited lower KPS scores, and had a higher prevalence of ECM.

**Table 1:** Baseline characteristics of patients by PIV group.

Characteristics	Low PIV (%)	High PIV (%)	*p*
*N*	1570	2286	
Age, years (mean (SD))	56.19 (11.50)	57.41 (12.02)	0.002
Gender, male (%)	790 (50.3)	846 (37.0)	<0.001
Extracranial metastases, yes = 1 (%)	987 (62.9)	1649 (72.1)	<0.001
KPS categories (%)			<0.001
90-100	586 (37.3)	514 (22.5)	
70-80	747 (47.6)	1342 (58.7)	
<80	237 (15.1)	430 (18.8)	
Primary cancer site (%)			<0.001
Breast cancer	69 (4.4)	61 (2.7)	
lung cancer	1027 (65.4)	1696 (74.2)	
Nasopharyngeal carcinoma	100 (6.4)	70 (3.1)	
Others	374 (23.8)	459 (20.1)	
Number of brain metastases (%)			0.068
1	506 (32.2)	658 (28.8)	
2 to 3	127 (8.1)	202 (8.8)	
>3	937 (59.7)	1426 (62.4)	
Chemotherapy, yes (%)	993 (63.2)	1407 (61.5)	0.300
Targeted therapy, yes (%)	479 (30.5)	658 (28.8)	0.263
Immunotherapy, yes (%)	31 (2.0)	62 (2.7)	0.174
Radiotherapy, yes (%)	721 (45.9)	974 (42.6)	0.045

### 3.2 Survival analysis

**Figure 1:**
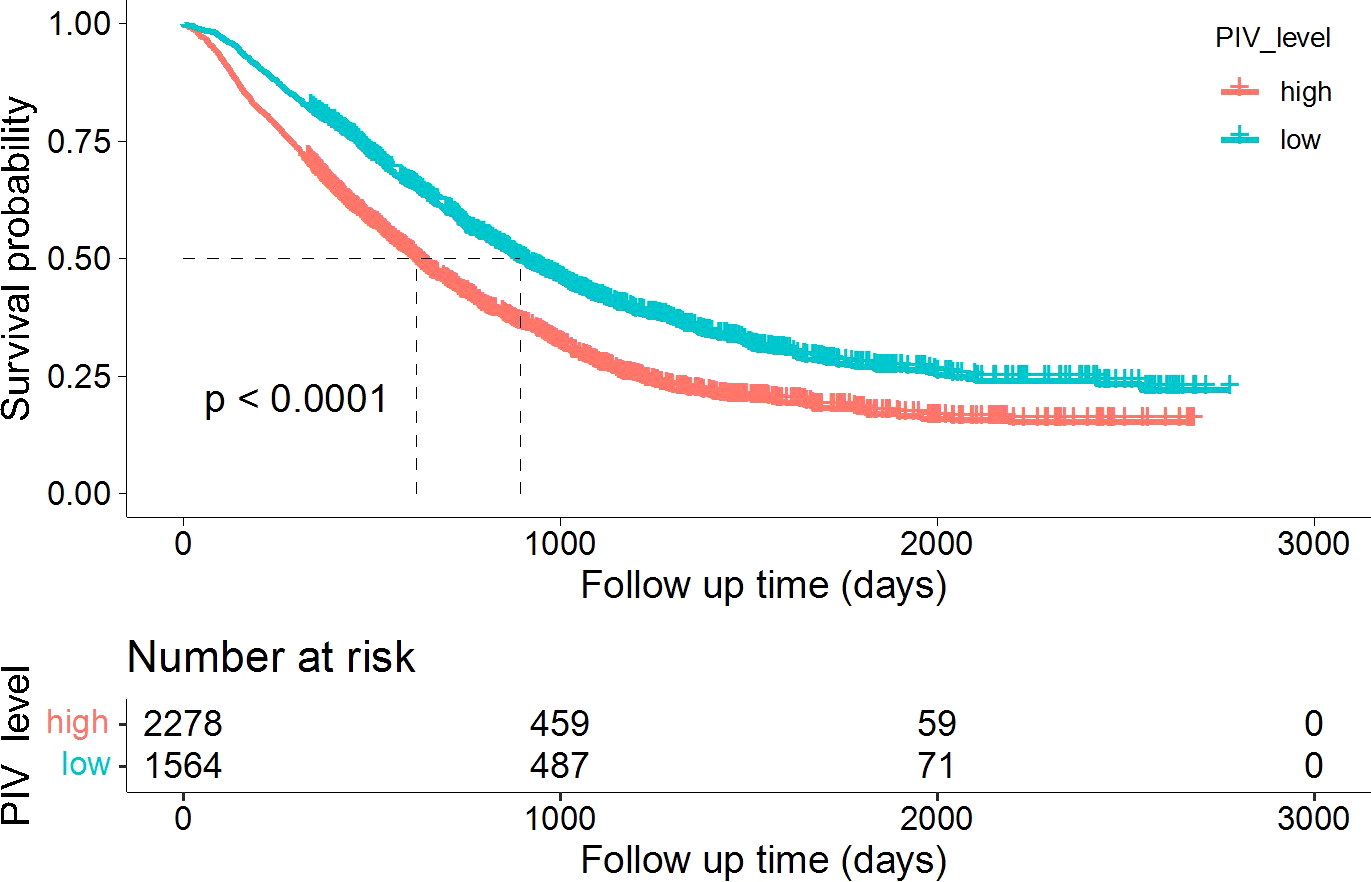
Kaplan-Meier survival analysis according to PIV in patients with brain metastases. Kaplan-Meier curves for overall survival (OS) stratified by PIV level. Patients with high PIV (red line) had significantly worse OS compared with those with low PIV (teal line, *p* < 0.0001). The number of patients at risk is shown below the curves.

**Figure 2:**
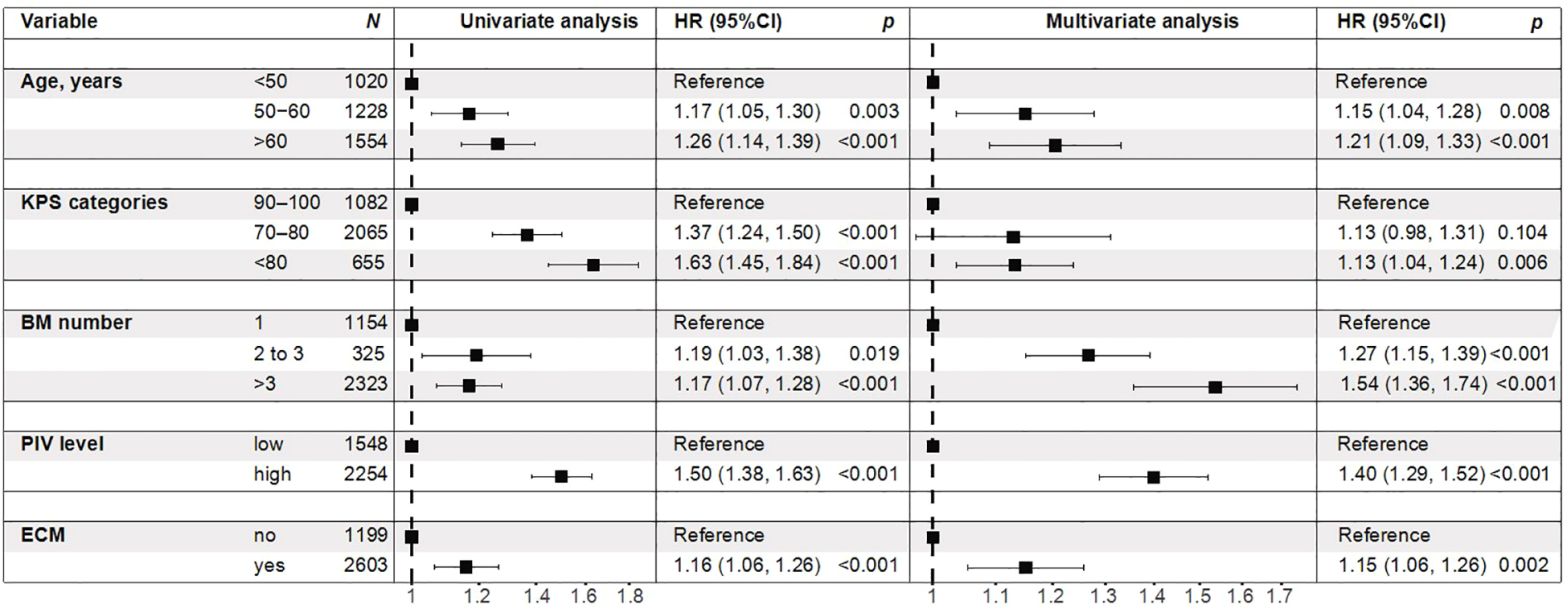
The Cox proportional hazards analyses of PIV level and GPA risk score with OS in patients with brain metastases. Multivariable model adjusted for age, KPS, number of brain metastases, and extracranial metastases. PIV, Pan-Immune-Inflammation Value; GPA, Graded Prognostic Assessment; BM, brain metastases; KPS, Karnofsky Performance Status; ECM, extracranial metastases; HR, hazard ratio; CI, confidence interval.

Kaplan-Meier survival analysis showed that patients with high PIV had significantly worse OS compared with those with low PIV (*p* < 0.001), indicating that elevated baseline PIV was associated with poorer prognosis (**Figure 1**). On univariate Cox regression, age, KPS score, number of brain metastases, PIV level, and extracranial metastases ECM were all significant prognostic factors for OS. In the multivariable model adjusted for age, KPS, number of brain metastases, and extracranial metastases, high PIV remained independently associated with increased mortality (HR 1.40, 95% CI 1.29-1.52; p<0.001) (**Figure 2**).

### 3.3 Nomogram construction

**Figure 3:**
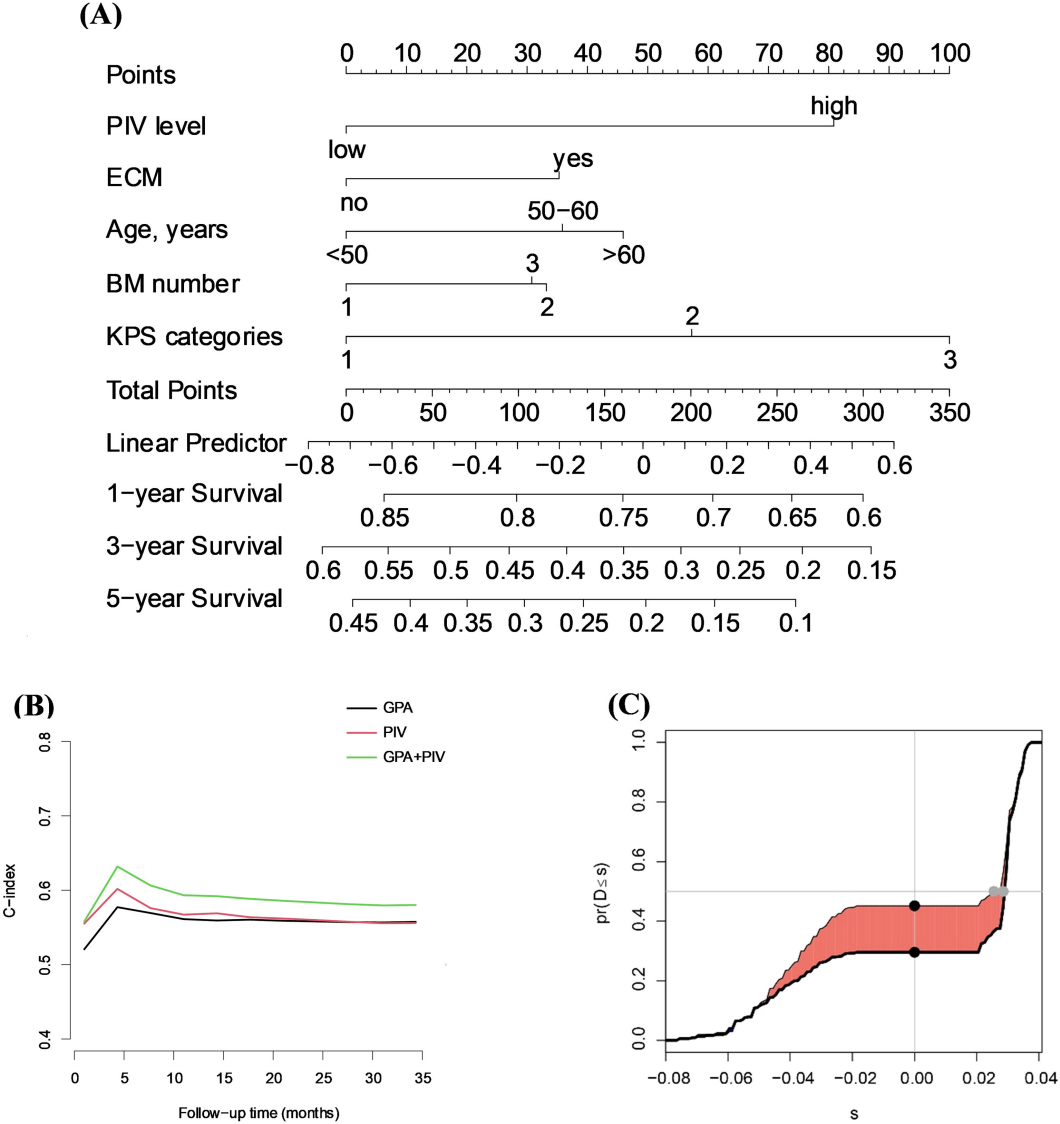
Development and validation of the prognostic model incorporating PIV. **(A)** Nomogram integrating PIV and clinical variables (extracranial metastases, age, brain metastasis number, and KPS categories) to predict 1-, 3-, and 5-year OS in patients with brain metastases. **(B)** Time-dependent C-index curves comparing the discriminative ability of the GPA model, PIV alone, and the combined GPA+PIV model. The combined model showed consistently higher predictive performance during follow-up. **(C)** Integrated Discrimination Improvement (IDI) analysis comparing the PIV-based model with the baseline model. The x-axis (s) indicates discrimination improvement (difference in predicted probabilities, no unit), and the y-axis [pr (D ≤ s)] shows its empirical cumulative distribution; the shaded area demonstrates a modest but statistically significant improvement with 95% CI not crossing zero.

We developed a prognostic nomogram that integrates PIV with key clinical variables, including extracranial metastases (ECM), age, number of brain metastases, and KPS categories, to estimate overall survival at one, three, and five years in patients with brain metastases (**Figure 3A**). The nomogram allows for the calculation of a total point score based on these predictors, which corresponds to a linear predictor value and subsequent survival probabilities.

### 3.4 Model discrimination and calibration

Time-dependent C-index analysis showed that the GPA+PIV model had higher discriminative performance throughout follow-up compared with the GPA model or PIV alone (**Figure 3B**). The addition of PIV resulted in a statistically significant improvement in discrimination (IDI 0.010; 95% CI 0.006-0.015; p<0.001) (**Figure 3C**). Calibration for 1-year survival demonstrated good agreement between predicted and observed probabilities (**Supplementary Figure S2**), with slight deviation in the highest-risk group.

### 3.5 Subgroup analyses

The association between elevated PIV and worse survival was consistent across treatment-defined subgroups (**Table 2**). In multivariable analyses, high PIV remained an independent risk factor among patients receiving or not receiving chemotherapy (HR 1.35 and 1.43), targeted therapy (HR 1.37 and 1.38), and radiotherapy (HR 1.44 and 1.29), all p<0.001.When stratified by primary tumor type, high PIV was associated with poorer survival in both lung cancer and other primary tumors in univariate and multivariable models (**Supplementary Figures S3, S4**).Sex-stratified analyses also demonstrated a significant association between high PIV and mortality in both females (HR 1.36, 95% CI 1.22-1.52) and males (HR 1.61, 95% CI 1.42-1.83), all p<0.001 (**Supplementary Figures S5, S6**). Interaction terms were explored, and no subgroup demonstrated effect reversal.

**Table 2:** Association between PIV and OS across treatment subgroups.

Subgroup	Treatment status	Univariate analysis		Multivariate analysis	
		HR (95% CI)	*p*	HR (95% CI)	*p*
Chemotherapy	Received	1.48 (1.34, 1.64)	<0.001	1.35 (1.22, 1.50)	<0.001
	Not Received	1.51 (1.32, 1.73)	<0.001	1.43 (1.24, 1.64)	<0.001
Targeted therapy	Received	1.52 (1.31, 1.77)	<0.001	1.37 (1.24, 1.51)	<0.001
	Not Received	1.49 (1.35, 1.64)	<0.001	1.38 (1.18, 1.61)	<0.001
Radiotherapy	Received	1.43 (1.27, 1.62)	<0.001	1.44 (1.29, 1.61)	<0.001
	Not Received	1.54 (1.38, 1.72)	<0.001	1.29 (1.14, 1.46)	<0.001

### 3.6 Sensitivity analyses

To evaluate the robustness of the prognostic association between PIV and overall survival, sensitivity analyses were performed using alternative representations of the biomarker. First, Cox regression models were repeated using the median PIV value as the dichotomization threshold. High PIV defined by the median remained significantly associated with inferior survival in both the univariate model (HR 1.44, 95% CI 1.33-1.56, p<0.001) and the multivariable model adjusted for age, KPS, number of brain metastases, and extracranial metastases (HR 1.35, 95% CI 1.25-1.47, p<0.001) (**Supplementary Figure S7**). Second, PIV was modeled as a continuous variable after Z-score standardization to avoid information loss from categorization. Each one-standard-deviation increase in PIV was associated with a higher mortality risk in both univariate (HR 1.13, 95% CI 1.10-1.15, p<0.001) and multivariable analyses (HR 1.11, 95% CI 1.08-1.14, p<0.001) (**Supplementary Figure S8**). Across both sensitivity approaches, effect sizes for PIV remained consistent with the primary analysis, and the direction of the PIV-survival association were stable.

## 4 Discussion

This study demonstrates that the PIV is an independent prognostic biomarker in patients with brain metastases and that integrating PIV with the GPA improves risk stratification beyond established clinical factors. These findings address a central challenge in the management of brain metastases, in which prognostic tools remain heavily dependent on static clinical parameters that do not fully capture patient-specific biological processes.

The biological plausibility of PIV as a prognostic marker is supported by the central role of systemic inflammation in tumor dissemination and treatment response (29, 30). As a composite index integrating neutrophils, monocytes, platelets, and lymphocytes, PIV reflects multiple coordinated components of the host immune response (31–33). These processes are relevant in brain metastasis, where the microenvironment is characterized by innate immune activation, microglial engagement, and chronic T-cell dysfunction (34, 35). These interactions characterize a host inflammatory state permissive for intracranial tumor progression. Although mechanistic pathways were not directly evaluated in this study, the ability of PIV to capture these integrated inflammatory processes provides a coherent rationale for its association with survival.

Our findings are consistent with prior work showing that inflammation-based biomarkers carry prognostic value across several malignancies (36–39). However, evidence specifically in brain metastases has been limited. By evaluating a large and heterogeneous cohort, this study expands the existing literature and supports the relevance of systemic inflammatory profiling in this unique clinical population.

The prognostic effect of PIV was preserved across all major treatment-defined subgroups, including patients receiving chemotherapy, targeted therapy, or radiotherapy. This consistency indicates that PIV reflects a systemic inflammatory phenotype that remains prognostically relevant regardless of treatment modality.

This consistency underscores the value of PIV as a generalizable biomarker across diverse therapeutic contexts. The association was also observed in analyses stratified by primary tumor types, indicating that PIV reflects a shared systemic inflammatory phenotype rather than a histology-specific mechanism. However, inflammatory microenvironments differ across cancers, and tumor-specific analyses will be needed to determine whether PIV interacts with histology-specific immune mechanisms (40). Placing PIV within the broader biomarker landscape, recent prognostic tools for brain metastases increasingly incorporate tumor-specific modalities such as circulating tumor DNA, radiomics signatures, and immune-related transcriptional classifiers (41–43). These biomarkers capture tumor-intrinsic or microenvironmental features, whereas PIV reflects systemic host inflammation (44, 45), the two perspectives are complementary rather than competing. Although PIV lacks the tumor-specific precision of genomic or imaging markers, its simplicity and biological breadth make it a useful foundational component within multimodal prognostic frameworks. Future studies incorporating larger tumor-specific cohorts and integrating molecular, inflammatory, and imaging biomarkers will help delineate whether PIV primarily reflects shared host biology or interacts with histology-specific mechanisms.

Sex-stratified analyses showed that the prognostic association of PIV was present in both men and women, with differences in effect magnitude. Although exploratory, this variation may relate to known sex-based differences in systemic immune regulation (46, 47). These subgroup results collectively demonstrate the generalizability of PIV across diverse clinical contexts.

Beyond subgroup consistency, the robustness of the prognostic association was further supported by sensitivity analyses. Using the median PIV as an alternative cutoff produced results highly consistent with the main model, indicating that the prognostic effect is not dependent on the specific threshold used for dichotomization. Moreover, modeling PIV as a standardized continuous variable yielded similar effect estimates, demonstrating that the association persists without categorization and reflects a graded relationship between systemic inflammatory burden and survival. These findings confirm that the prognostic relevance of PIV is stable across analytic specifications and strengthens confidence in its biological and clinical validity.

Integration of PIV with the GPA produced meaningful improvements in risk discrimination and supported the development of a clinically accessible nomogram. Calibration analysis demonstrated overall good agreement between predicted and observed survival, with a minor tendency toward overestimation in the highest-risk group, a pattern commonly seen in prognostic models derived from heterogeneous clinical cohorts. Although the incremental improvement in discrimination provided by adding PIV to the GPA was modest in absolute magnitude, this degree of enhancement is consistent with expectations when incorporating a single biologically grounded marker into an established multivariable framework. The stability of the association between PIV and survival across analytic approaches further supports its role as a meaningful independent prognostic factor. Taken together, these findings indicate that PIV provides complementary biological information that enhances the performance of the GPA and strengthens its utility within a combined prognostic model.

Beyond statistical enhancement, these findings have practical implications. Patients with markedly elevated PIV may warrant closer surveillance, earlier imaging, or more proactive local therapy. Because lymphopenia and myeloid expansion have been associated with reduced response to immunotherapy, PIV may help identify patients who require alternative systemic approaches rather than immunotherapy alone. The simplicity, low cost, and repeatability of PIV raise the possibility of its use in longitudinal monitoring to detect inflammatory shifts associated with tumor progression or therapeutic response. The GPA+PIV model enabled clearer separation of high- and low-risk groups and facilitated the construction of a clinically accessible nomogram, which may help clinicians better individualize treatment decisions in routine practice.

This study has limitations. Its retrospective, single-center design may introduce selection bias and limit generalizability. Residual inflammatory confounding cannot be fully excluded despite standardized timing of hematologic measurements, as data on corticosteroid use, metastasis volume, and neurosurgical interventions were unavailable and may introduce additional bias. External validation in independent cohorts will be necessary to confirm the robustness of the proposed model, and longitudinal assessments of PIV may clarify whether dynamic changes carry additional prognostic value. Finally, because PIV reflects systemic rather than tumor-specific biology, integration with molecular or imaging biomarkers may enhance prognostic precision.

Despite these limitations, our findings indicate that PIV provides meaningful prognostic information as an independent biomarker while also enhancing the performance of established clinical tools. Future research should prioritize external validation in multi-institutional cohorts and studies incorporating dynamic longitudinal assessment of PIV to clarify its dynamic prognostic value. In addition, future studies with serial hematologic monitoring may help better characterize how treatment-induced fluctuations interact with PIV trajectories over time. Integrating PIV with molecular and imaging-based biomarkers may ultimately lead to more comprehensive and biologically informed prognostic models.

In conclusion, PIV captures host systemic inflammatory activity that is not reflected in conventional clinical parameters and provides meaningful prognostic information in patients with brain metastases. Its consistent performance across clinical subgroups, stability across analytic approaches, and ability to improve GPA-based risk stratification support its use as a complementary prognostic indicator. Prospective multicenter validation incorporating dynamic longitudinal assessment of PIV and multimodal biomarkers will be essential to define its broader clinical applicability.

## Data Availability

The raw data supporting the conclusions of this article will be made available by the authors, without undue reservation.
