# Supplementary material for: Pan-immune-inflammation value as an independent prognostic marker in patients with brain metastases
Source: Front Oncol. 2026 Jan 6;15:1718288. doi: 10.3389/fonc.2025.1718288 (PMC12815847; doi:10.3389/fonc.2025.1718288)
Supplement: Supplementary file 1 [file Presentation1.pdf]

Supplementary Material

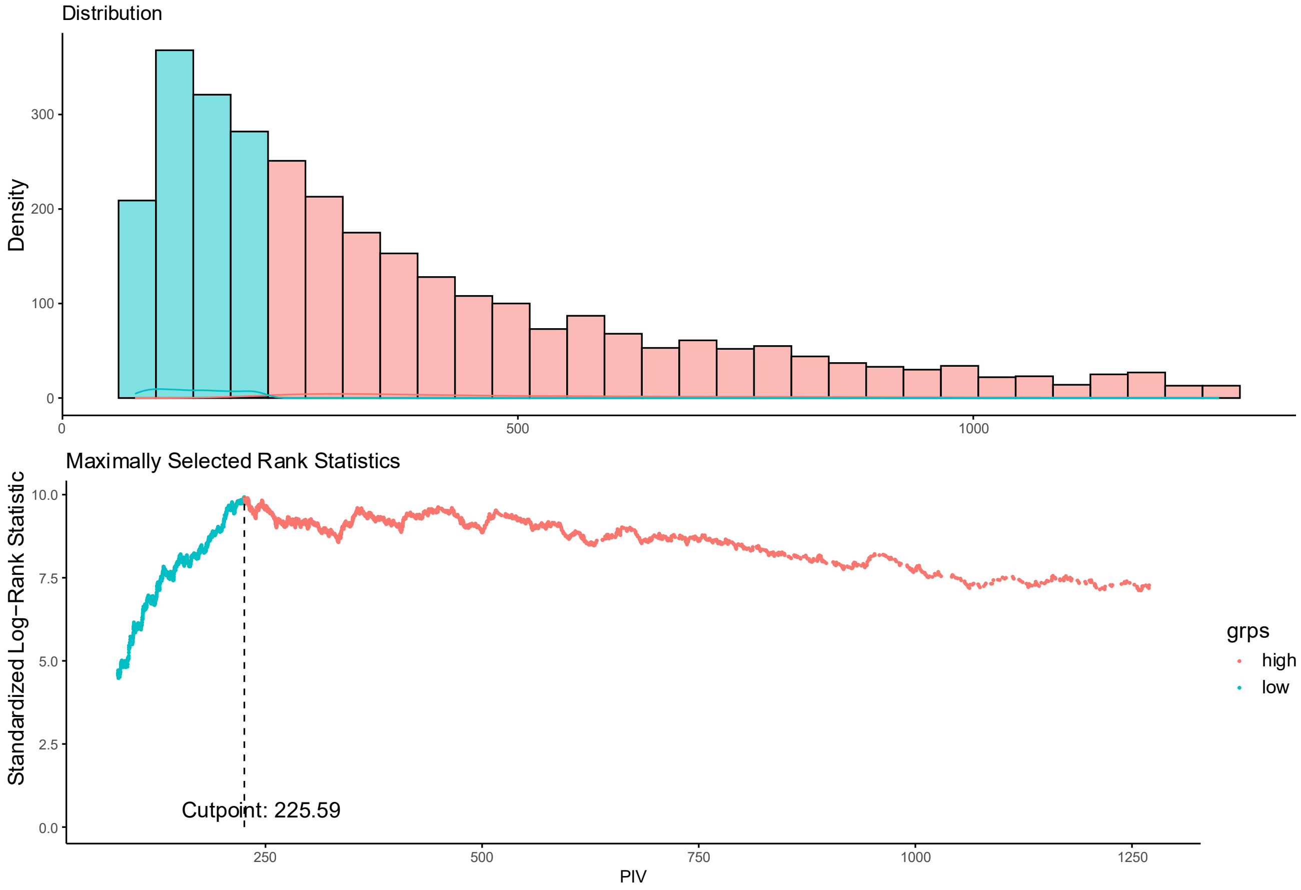

Supplementary Figure 1 Determination of the optimal cutoff value for the pan-immune-inflammation value (PIV). The upper panel shows the distribution of PIV in the study cohort. The lower panel depicts the standardized log-rank statistics across a range of cutoff points, with the maximum value corresponding to the optimal cutoff. The cutoff value was identified as 225.59, which was used to stratify patients into low- and high-PIV groups.

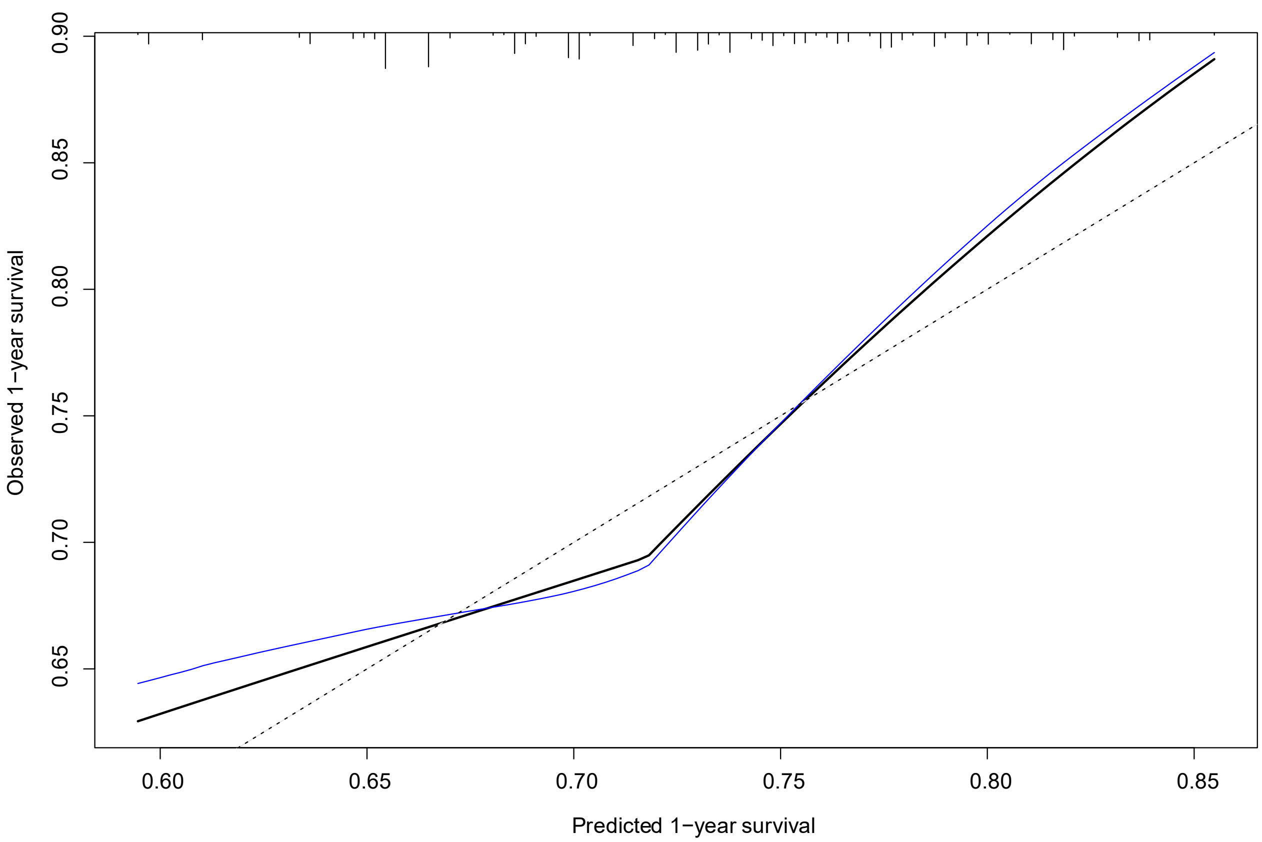

Supplementary Figure 2 Calibration plot for 1-year overall survival of the GPA+PIV model. The x-axis represents the model-predicted probability of 1-year survival, and the y-axis represents the observed survival estimated by Kaplan-Meier analysis. The solid line indicates the performance of the GPA+PIV model, while the dashed line represents the ideal reference line where predicted survival would perfectly match the observed outcome. The plot shows good agreement between predicted and observed probabilities, with slight overestimation in the highest-risk group.

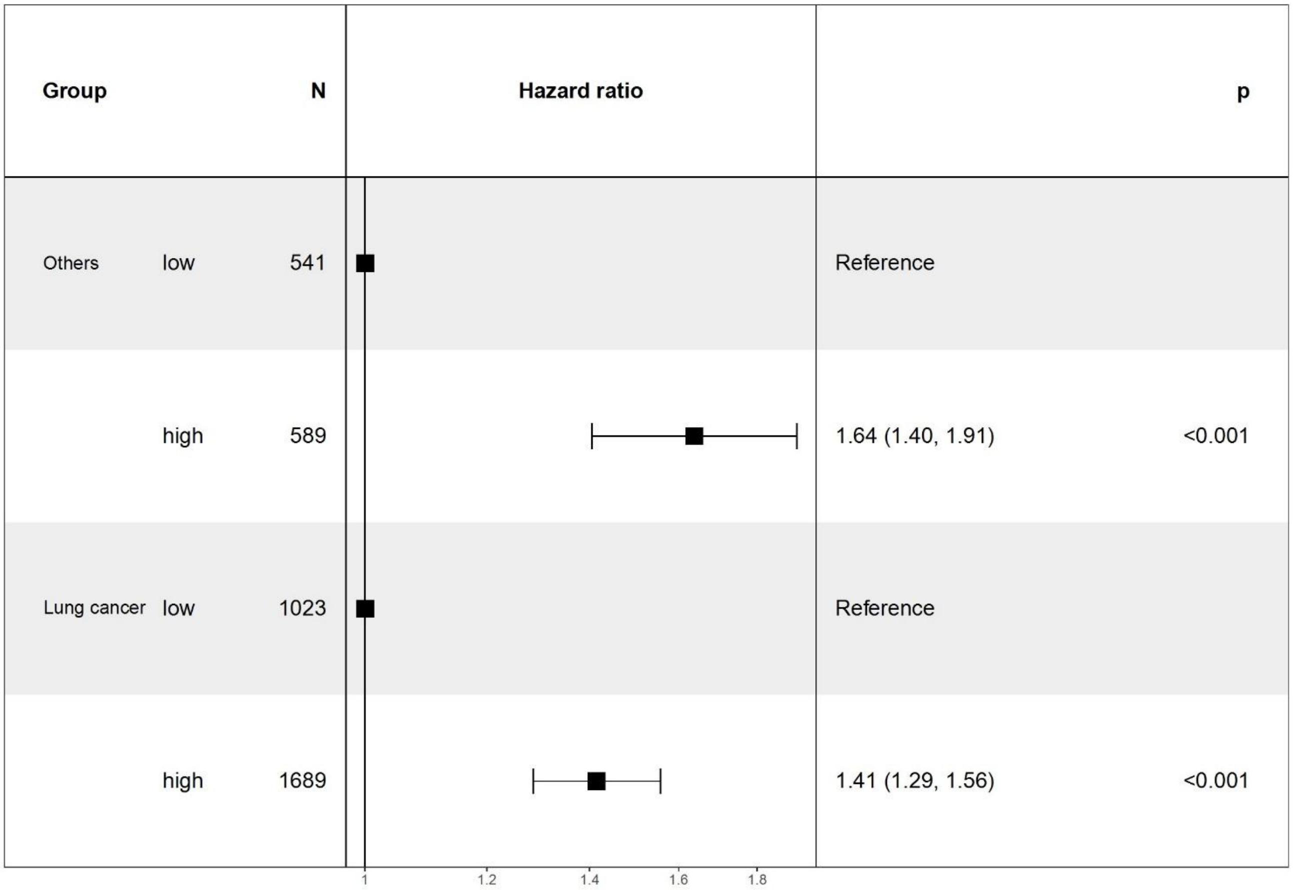

Supplementary Figure 3 Subgroup analysis of overall survival according to primary cancer. Univariate Cox regression forest plot illustrating the prognostic impact of pan-immune-inflammation value (PIV). Compared with the low-PIV group, high PIV was significantly associated with shorter overall survival in both other cancers (HR = 1.64, 95% CI: 1.40-1.91, $p < 0.001$) and lung cancer (HR = 1.41, 95% CI: 1.29-1.56, $p < 0.001$).

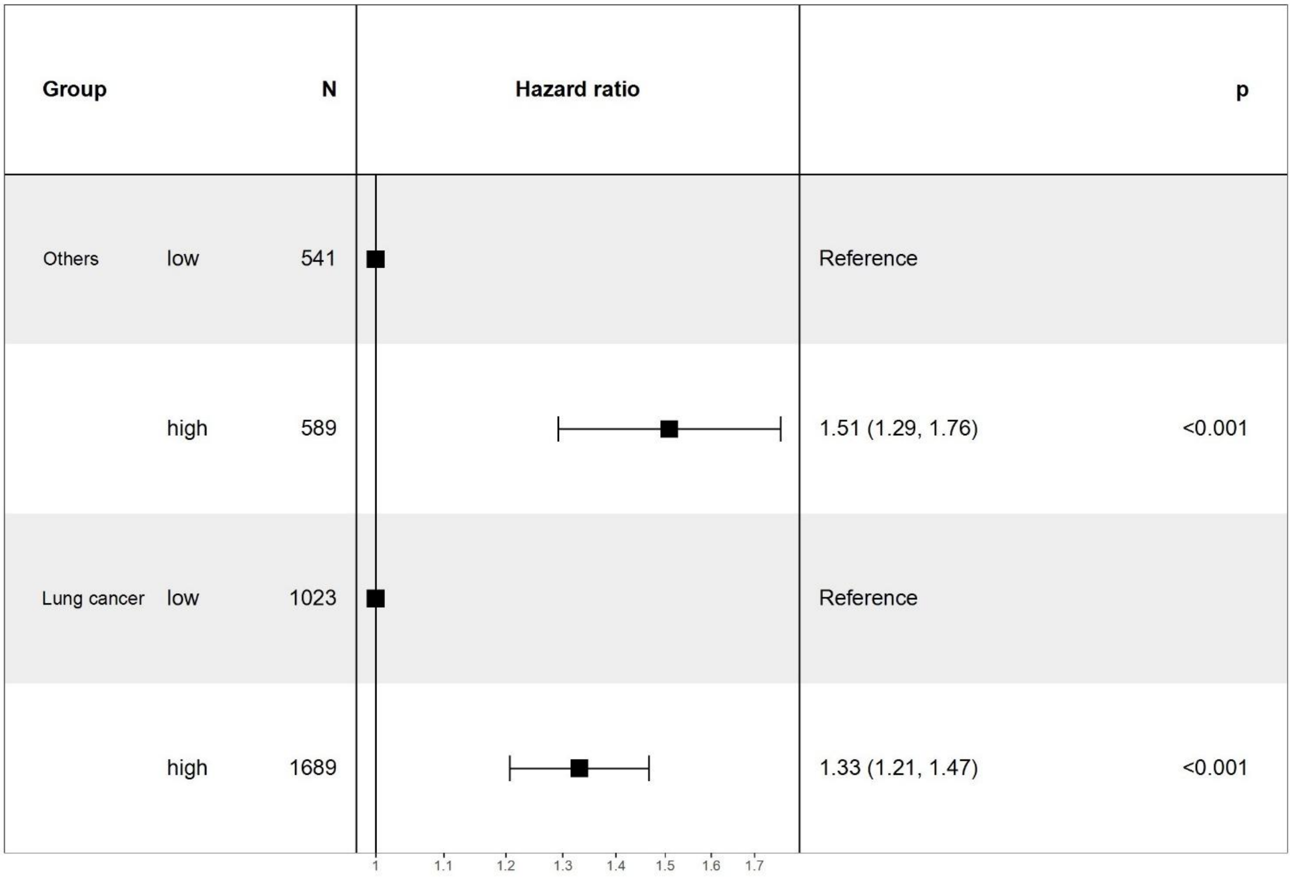

Supplementary Figure 4 Subgroup analysis of overall survival according to primary cancer. Multivariate Cox regression forest plot showing the prognostic impact of pan-immune-inflammation value (PIV) after adjustment for baseline clinical factors. Compared with the low-PIV group, high PIV was significantly associated with shorter overall survival in both other cancers (HR = 1.51, 95% CI: 1.29-1.76, $p < 0.001$) and lung cancer (HR = 1.33, 95% CI: 1.21-1.47, $p < 0.001$).

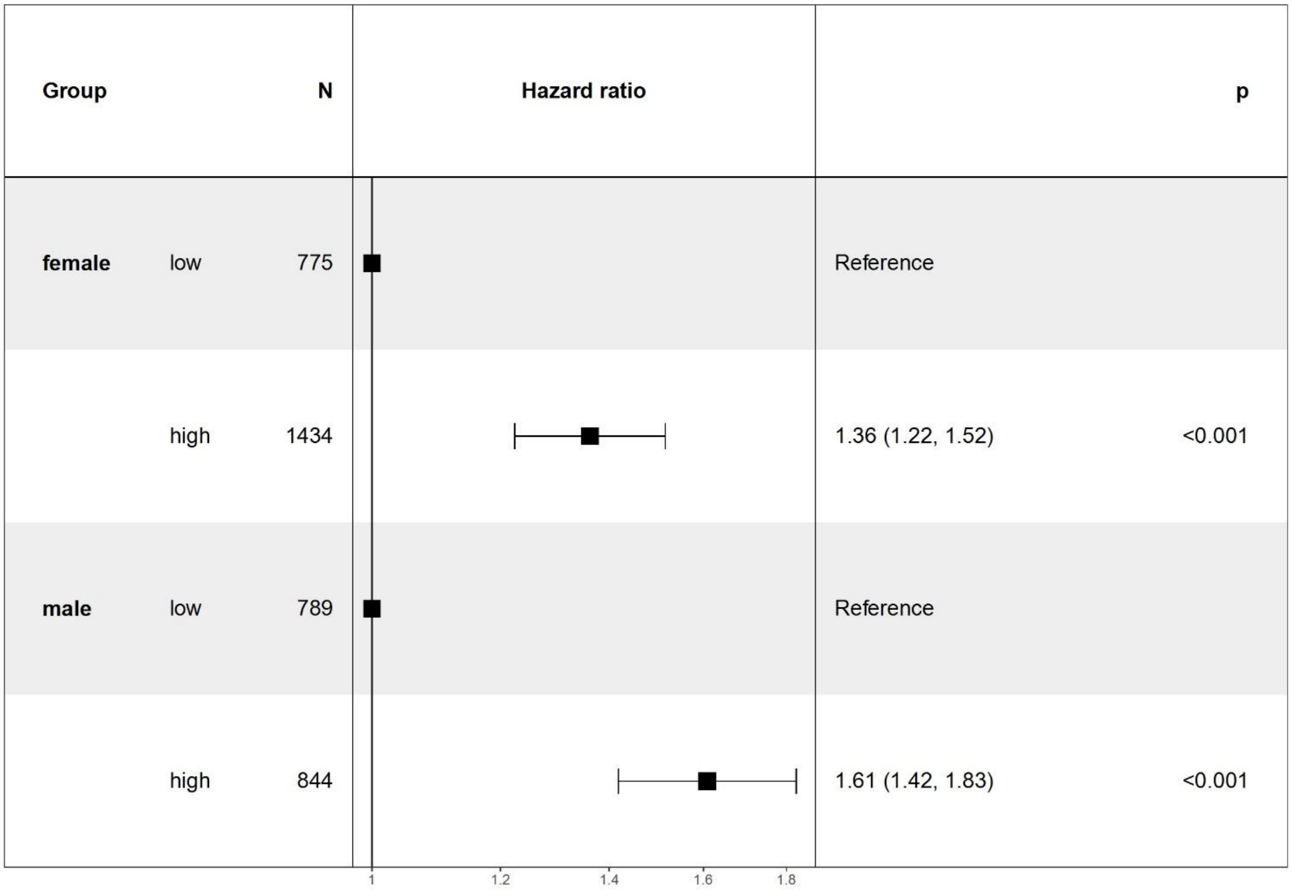

Supplementary Figure 5 Subgroup analysis of overall survival according to sex. Univariate Cox regression forest plot illustrating the prognostic impact of pan-immune-inflammation value (PIV). Compared with the low-PIV group, high PIV was significantly associated with shorter overall survival in both female (HR = 1.36, 95% CI: 1.22-1.52, p < 0.001) and male (HR = 1.61, 95% CI: 1.42-1.83, p < 0.001).

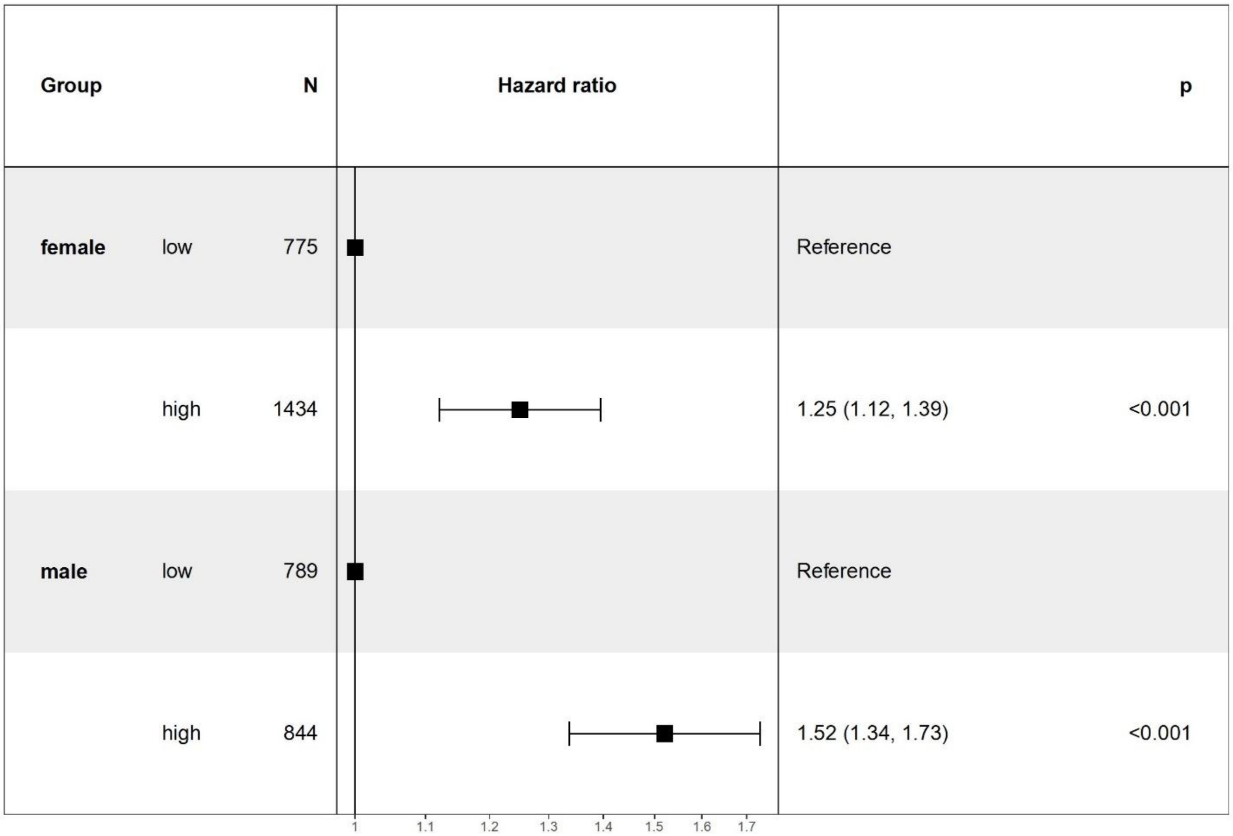

Supplementary Figure 6 Subgroup analysis of overall survival according to sex. Multivariate Cox regression forest plot showing the prognostic impact of pan-immune-inflammation value (PIV) after adjustment for baseline clinical factors. Compared with the low-PIV group, high PIV was significantly associated with shorter overall survival in both other cancers (HR = 1.25, 95% CI: 1.12-1.39, $p < 0.001$) and lung cancer (HR = 1.52, 95% CI: 1.34-1.73, $p < 0.001$).

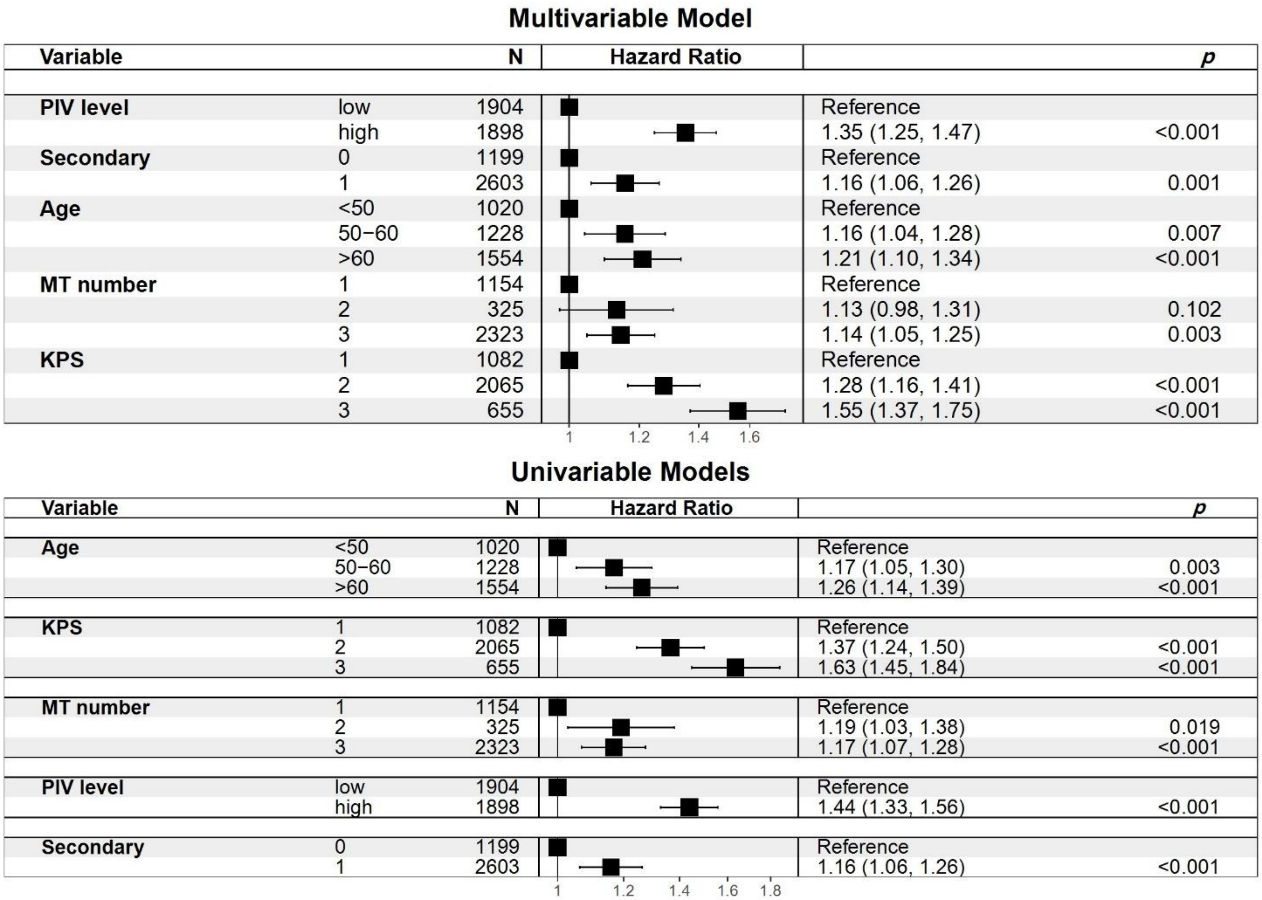

Supplementary Figure 7 Sensitivity analysis using median PIV cutoff: univariate and multivariate Cox regression models. Forest plots display hazard ratios (HRs) and 95% confidence intervals (CIs) for overall survival when the pan-immune-inflammation value (PIV) is dichotomized using the cohort median as an alternative cutoff. The upper panel shows the multivariable Cox model adjusted for age, Karnofsky Performance Status (KPS), number of brain metastases, and extracranial metastases. The lower panel presents the corresponding univariate Cox models. Across both analytic approaches, high PIV remained significantly associated with increased mortality, demonstrating that the prognostic effect of PIV is robust to changes in cutoff definition.

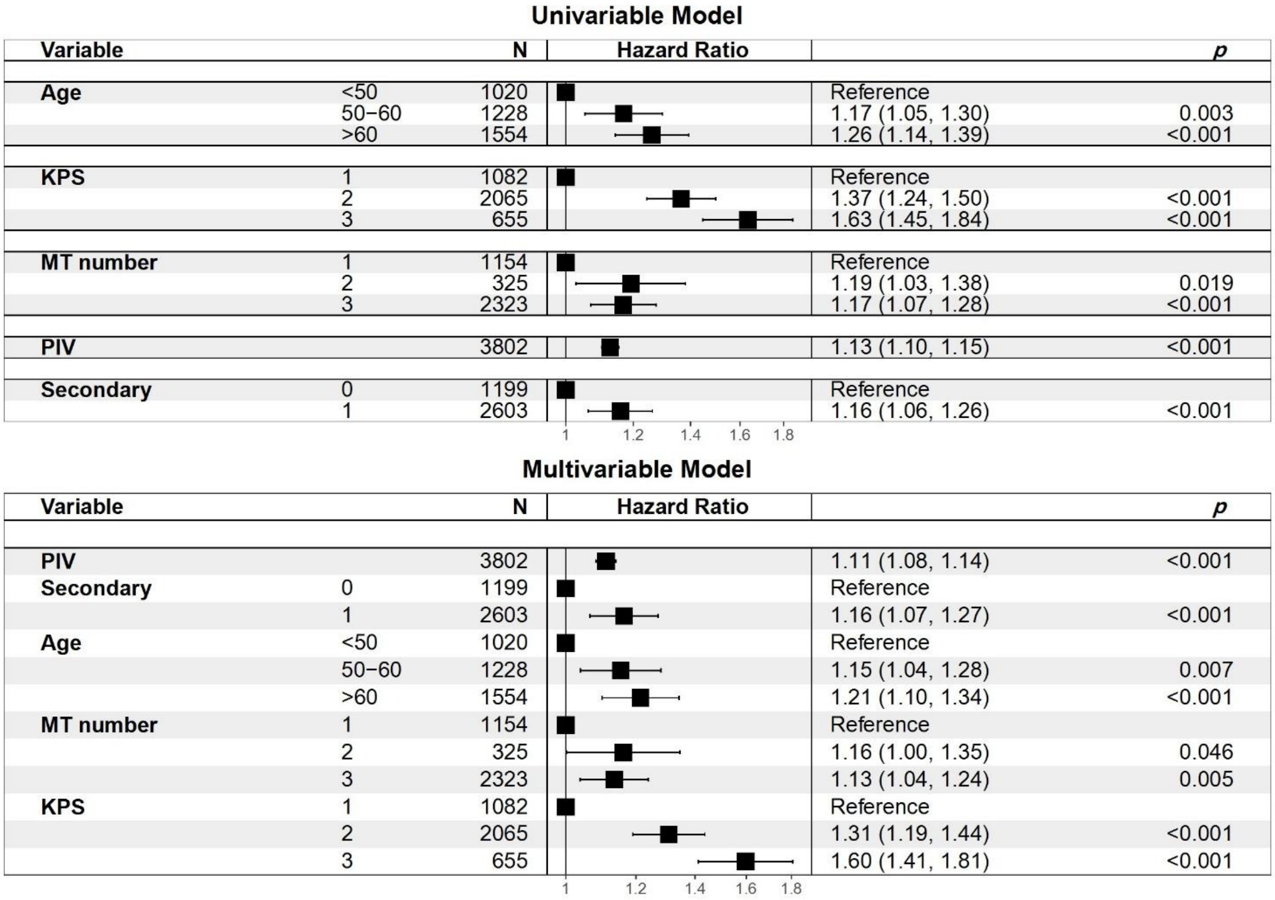

Supplementary Figure 8 Sensitivity analysis using PIV as a standardized continuous variable: univariate and multivariable Cox regression models. Forest plots show hazard ratios (HRs) and 95% confidence intervals (CIs) for overall survival when the pan-immune-inflammation value (PIV) is modeled as a continuous variable after Z-score standardization. The upper panel presents univariate Cox regression results, and the lower panel displays the multivariable Cox model adjusted for age, Karnofsky Performance Status (KPS), number of brain metastases, and extracranial metastases. Each one-standard-deviation increase in PIV remained significantly associated with higher mortality in both models, demonstrating that the prognostic effect of PIV persists without categorical grouping and is robust to the modeling approach.
